# Diagnosis and Management of Gastro-pleural Fistula in Metastatic Malignancy

**DOI:** 10.7759/cureus.4455

**Published:** 2019-04-13

**Authors:** Nadia Baka, Vivek Batra, Vincent Yeung, Shuwen Lin

**Affiliations:** 1 Miscellaneous, Sidney Kimmel Medical College at Thomas Jefferson University, Philadelphia, USA; 2 Medical Oncology, Thomas Jefferson University Hospital, Philadelphia, USA; 3 Internal Medicine, Thomas Jefferson University Hospital, Philadelphia, USA

**Keywords:** fistula, cancer, pleura, stomach, empyema, tki, conservative treatment, metastatic disease

## Abstract

Gastro-pleural fistula is a rare condition, and the diagnosis can be challenging, as demonstrated in our case. The management is even more complex, with wide gamut of management strategies from more conservative management such as endoscopic closures and minimally invasive video-assisted thoracoscopic surgery (VATS) to open surgical repair. We present the case of a 55-year-old female with metastatic renal cell cancer with prior radiation therapy and cabozantinib treatment who was diagnosed with gastro-pleural fistula after extensive workup. She underwent endoscopic closure and subsequent jejunostomy tube feeding, venting gastrostomy tube, and draining chest tube. Antibiotics and chest tube drainage were primary modalities for treatment of her empyema. Subsequently, she required laparoscopic surgery for fistula repair.

## Introduction

Markowitz and Herter described two case reports in 1959, in their seminal paper, wherein they proposed that gastro-pleural fistula is essentially a complication of esophageal hiatal hernia [1]. Their proposed etiologies of the condition include (1) perforation of an intra-thoracic stomach into the esophageal hernia, (2) trauma from acute injury, and/or (3) perforation of the stomach within the peritoneal cavity with secondary abscess formation and erosion through the diaphragm [1].

The fistulous communication between the stomach and the pleura can present in a rather challenging manner, and clinical diagnosis is not straightforward. Symptoms can include dyspnea, fevers, scapular pain, pleurisy, chest pain, and cough. The disease usually presents acutely, but in some cases, it can be more indolent if the fistula is contained and small in size. Hiatal hernia, malignancy, chemotherapy, and radiation are risk factors for the development of the fistulous connection between the stomach and pleura. Imaging modalities such as cross-sectional computed tomography (CT), upper gastro-intestinal series (GI series), and magnetic resonance imaging (MRI) are primary diagnostic techniques. Evaluation of fistula closure, conversely, requires more dynamic testing, such as a dye test.

## Case presentation

A 55-year-old female with a history of renal cell carcinoma of the left kidney metastatic to the bony pelvis, lungs, mediastinum, and spleen presented to the emergency department with shortness of breath, pleuritic chest pain, and left scapular pain. She presented to the same emergency department one week prior with pleuritic chest pain but was discharged home after pulmonary embolism was ruled out. 

She was diagnosed with renal cell carcinoma of the left kidney five years prior after presenting with gross hematuria. At that time, she underwent left radical nephrectomy. One year later, she developed a metastatic lesion in the bony pelvis for which she underwent radiation therapy. She as treated with pazopanib for two years with stable disease but stopped due to gastro-intestinal toxicity. Therapy was switched to nivolumab, which was discontinued after six months due to grade four pancreatitis and grade two rash. Eight months prior to her current presentation, she underwent radiation treatment to metastatic lesions in the left pubic symphysis and spleen. The patient initiated therapy with cabozantinib, a tyrosine-kinase-inhibitor used to treat renal cell carcinoma, three months prior to her current presentation. 

On physical examination, she was wheezing in all lung fields and hypoxemic requiring supplemental oxygen. She had prior 12-pack-year smoking history but no formal diagnosis of chronic obstructive pulmonary disease (COPD). A chest x-ray revealed a small left pleural effusion and left basilar atelectasis. Laboratory workup, including complete blood count, renal and hepatic panels, and troponin, was unremarkable. An electrocardiogram (ECG) revealed sinus tachycardia without signs of ischemia. CT was not repeated due to her negative CT angiogram one-week prior. Given radicular and left scapular pain, an MRI of the spine was done, which revealed no pathologic metastases in the thoracic or lumbar spine but did reveal a new sacral lesion. Given her progressive stridor, she underwent laryngoscopy, which revealed a normal upper airway. A bronchoscopy showed significant trachea-bronchomalacia and thick purulent secretions in the left upper lobe, lingula, and right upper lobe. 

**Figure 1:**
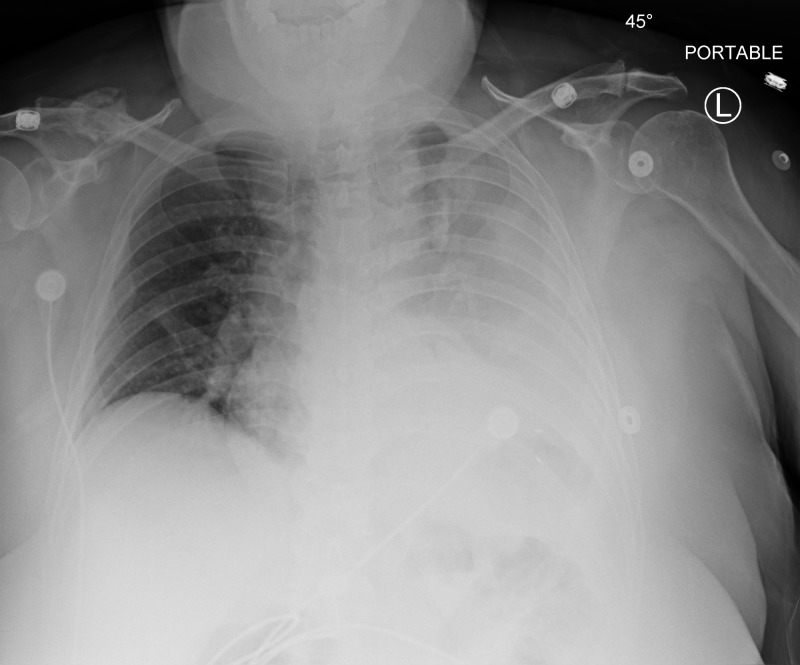
Repeat chest X-ray two days after admission reveals near-complete opacification of the left hemithorax

Two days after admission, repeat chest X-ray revealed near complete opacification of left lung and large pleural effusion, a remarkably different radiograph from admission (Figure 1).

Subsequent CT chest revealed a large left pleural effusion with partial loculation as well as partial atelectasis of the left upper lobe and complete atelectasis of the left lower lobe. A right perihilar metastasis and perisplenic metastases were reported. The study was negative for pulmonary thromboembolism. 

**Figure 2:**
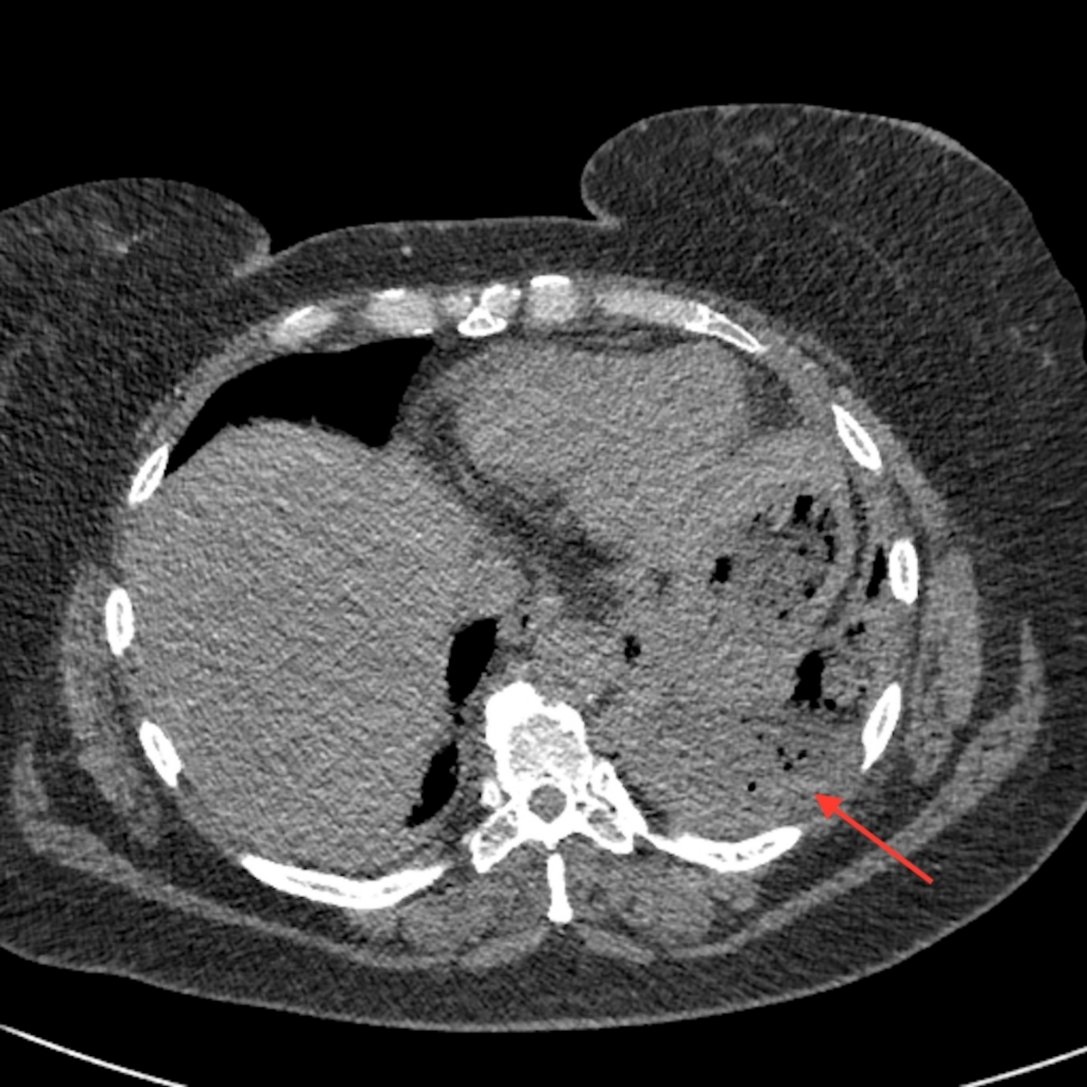
CT chest without contrast performed six days after chest tube placement reveals a collection of mottled foci of gas in the left upper quadrant below the diaphragm (arrow), representing a sub-phrenic abscess and/or a trans-diaphragmatic pleural fistula CT: computed tomography

Thoracentesis revealed cloudy straw colored exudative effusion. A four French pigtail catheter was placed. Approximately 400 milliliters of yellow-green fluid was immediately drained. Pleural fluid studies revealed a white blood cell count of 33,000/μL (97% neutrophils), pH of 6.44, LDH of 4760 U/L, and an amylase of 394 U/L. She was started on vancomycin, cefepime, and metronidazole for presumed empyema. Pleural fluid cultures showed heavy growth of *lactobacillus* species, heavy growth of anaerobic gram negative cocci, and moderate growth of *Candida krusei*. Antimicrobial therapy was subsequently narrowed to ertapenem and anidulafungin. Given lack of improvement and continued significant chest tube output over the following week, further CT imaging was obtained, revealing a gastro-pleural fistula (via the left diaphragm and superior posterolateral stomach) with associated complex pleural effusion containing contrast material and gas (Figure 2). This process abutted the known splenic metastases.

**Figure 3:**
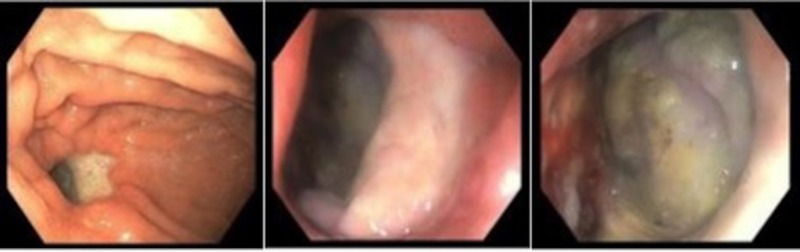
Images captured during the esophagogastroduodenoscopy reveal the defect in the stomach and fistula communicating with the pleural space

An esophagogastroduodenoscopy (EGD) revealed a 1.5-cm fistula in the posterolateral stomach that opened to the pleural space (Figure 3).

**Figure 4:**
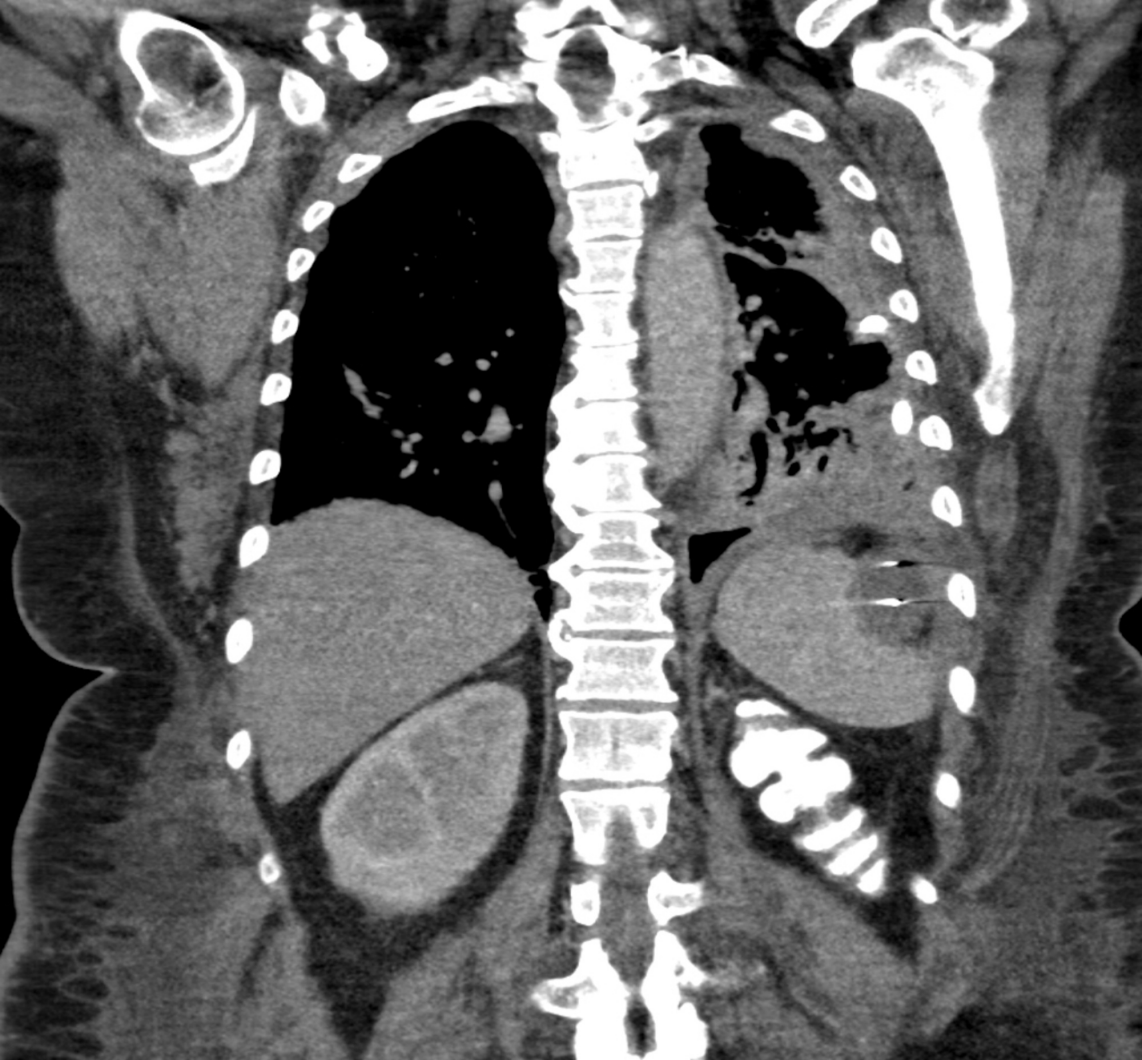
CT scan with oral contrast performed one day after esophagoduodenoscopy with endoscopic suturing of the fistula shows near-complete drainage of the left sub-diaphragmatic collection with residual gas There is no definite leakage of oral contrast into the left sub-diaphragmatic or pleural spaces. CT: computed tomography

Endoscopic suturing was attempted to close the fistula with limited success (partial closure noted on imaging, with methylene blue dye taken via mouth visualized in the chest tube drainage catheter on water seal; Figure 4). 

For complete closure, the authors attempted a novel approach utilizing a venting gastrostomy tube and chest tube to water seal to facilitate closure of the fistula over the ensuing six weeks. Enteral feeding via jejunostomy tube to aid closure of the fistula was employed. The patient was continued on ertapenem and anidulafungin. She was also initiated on a proton pump inhibitor. She was discharged to a rehabilitation facility with plans to repeat imaging and methylene blue swallow in six weeks.

**Figure 5:**
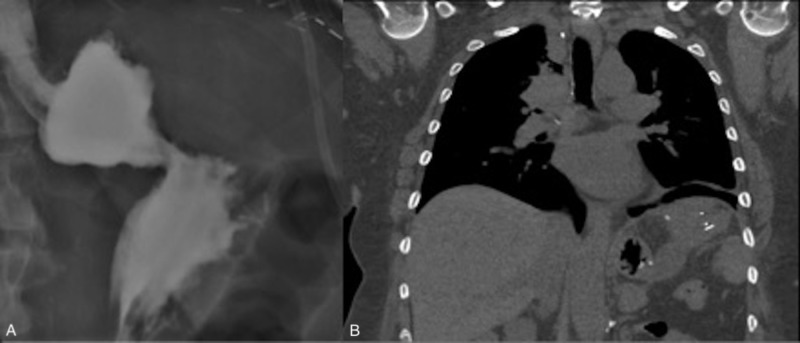
(A) Fluoroscopic upper GI series with oral contrast on post-operative day three shows no leakage of contrast outside of the stomach; (B) CT scan on post-operative day five shows no evidence of communication between the stomach and the pleural space GI: gastrointestinal, CT: computed tomography

Unfortunately, CT scans after six weeks showed that the fistula remained patent. A second attempt was made at endoscopic closure, which was again unsuccessful. One month later, during a hospitalization for electrolyte abnormalities, the patient decided to pursue elective surgical repair of the fistula in hopes of regaining the ability to resume normal oral intake. Four months after her initial presentation, she underwent laparoscopic surgery for fistula repair. The surgeon visualized extensive radiation fibrosis involving the stomach, spleen and retro-peritoneum. Given these findings and to avoid splenic bleeding, they pursued a conservative surgery whereby they stapled the stomach to ligate the gastro-pleural fistula anatomically. This approach is novel and was successful in our patient. A fluoroscopic upper GI series with oral contrast three days after surgery demonstrated no leakage of contrast outside of the GI tract or into the pleural space, and CT five days after surgery revealed no evidence of communication between the stomach and pleural space (Figure 5).

She tolerated an oral diet. Gastrostomy tube, jejunostomy tube, and chest tube were removed without complication.

## Discussion

Aero-digestive fistulas are a rare complication of a variety of medical conditions including infection, malignancy and its therapies, and surgery. They can occur in all age groups. Prognosis varies, with some patients making full recovery and others rapidly declining and succumbing to the complications.

Review of the literature reveals that most cases of aero-digestive fistulas occur in patients with one or more of the following risk factors: surgery and/or trauma to the gastro-intestinal tract or abdomen, perforated gastric ulcers, a history of recurrent or prolonged pulmonary infections, malignancy, and radiation therapy [1-13]. These conditions function to weaken the surrounding tissue, hence predisposing it to damage and fistulization. Studies have also shown a correlation between tyrosine kinase inhibitor use (particularly cabozantinib, sunitinib, sorafinib, and lenvatinib) and fistula formation [7,13]. Phase I and III trials of cabozantinib did not reveal fistula formation to be an observed adverse effect of this therapy [14-15]. Our patient’s risk factors included metastatic cancer, radiation therapy to the spleen and a three-month history of cabozantinib use. In addition, she had had previous abdominal procedures including a left radical nephrectomy.

Common presentations prompting workup included worsening respiratory symptoms, chest pain, shoulder pain, and food present in a chest tube [3,7,9-10,12]. If tachycardia and tachypnea are present, patients may inadvertently receive cardiac workup instead of a gastrointestinal workup, thereby delaying diagnosis [7]. 

Diagnostic workup consists of a wide variety of tests. This is often prompted by events such as food seen in a chest tube or chest tube drainage studies that are consistent with gastric secretions [12]. Imaging modalities that can reveal an aero-digestive fistula include contrast-enhanced CT, barium swallow, gastrografin study, or an upper GI series [2-3,6-7,9-12]. Methylene blue swallow is also utilized, with the presence of methylene blue dye in the chest tube diagnostic of fistulization [1]. On rare occasions, an endoscopy, thoracotomy, or exploratory laparotomy is necessary for diagnosis [1,5-6,16]. 

Management of gastro-digestive fistulas begins conservatively, with interventions such as antibiotics, bowel rest, and chest tube placement. In one study, four of six patients with fistulas were successfully managed conservatively. However, these four fistulas were small, asymptomatic, and incidentally found [2]. Conservative management nearly always fails, necessitating surgical management. Surgical interventions can include both thoracotomies and laparotomies and occasionally requires tissue resection (lung, spleen, stomach, and/or diaphragm) in addition to fistula repair [1,3,5-7,9-12]. There is one case of a successful repair using an esophageal stent [3]. Despite aggressive management, however, many patients will succumb to the complications of their disease [1,9,12].

Given the extent of her metastatic disease, we initially hoped to correct our patient’s defect by conservative measures. Unfortunately, both attempts to close the fistula via endoscopic suturing failed. Subsequently, we hoped the presence of a chest tube and bowel rest would reduce irritation to the area, allowing for spontaneous closure. Despite these measures, the fistula persisted 3 months later. Surgery was then pursued to afford the patient a better quality of life. This was successful, with complete closure of the fistula confirmed by upper GI series six weeks post-surgery.

## Conclusions

Gastro-pleural fistula is a rare complication of malignancy and its treatments. Diagnosis is difficult and often delayed. It is important to include gastro-pleural fistula in the differential for patients with risk factors along with other common primary cardiac and pulmonary pathologies, especially if the patient has tachycardia or tachypnea, as did our patient. Furthermore, we were focused on the possibility that her scapular pain was caused by a new metastasis. During the ensuing workup, we recognized that it was rather referred pain from peri-diaphragmatic pathology.

We had hoped to use conservative measures to treat this patient given the presence of metastatic cancer and past radiation therapy to the site of the defect would make for a difficult and tenuous surgery. Despite our attempts to treat this patient conservatively to facilitate spontaneous closure, the fistula persisted and we had to resort to surgical intervention to allow the patient a better quality of life.
